# Making a distinction between data cleaning and central monitoring in clinical trials

**DOI:** 10.1177/1740774520976617

**Published:** 2021-03-02

**Authors:** Sharon B Love, Victoria Yorke-Edwards, Carlos Diaz-Montana, Macey L Murray, Lindsey Masters, Michelle Gabriel, Nicola Joffe, Matthew R Sydes

**Affiliations:** MRC Clinical Trials Unit at UCL, London, UK

‘Data cleaning’ and ‘central monitoring’ have become intertwined to the potential detriment of trial conduct. They are practically and conceptually different. What is data cleaning, what is central monitoring and why does the difference matter?

Early clinical trials collected data on punch cards and then on paper. As computers became accessible, trialists began to enter data into a database towards the end of a trial and cleaned it before the analysis. As data started being entered centrally into computer databases on receipt of forms, trialists recognised that it was better to clean the data in real time. Many considered double data entry to reduce the amount of data cleaning.^1^ Now, with increasing use of electronic data capture to replace paper forms, staff at trial sites are entering data directly into databases and are prompted in real time with automated data checks. Further data cleaning is led centrally, often by trial managers and statisticians, and is achieved through checking against prescriptive or plausible ranges, by checking for logical sequences of events, and by checking that critical data (‘key variables’) are not missing. Van den Broeck and colleagues offer some advice on best practice for data cleaning.^2^

Monitoring of trials began with 100% source data verification – double-checking that the data on case report forms matched the patient’s hospital notes – and process checking at on-site monitoring visits. This required many dedicated monitors combing through hospital notes. Trials with more modest budgets conducted source data verification on only a sample of participants or a subset of datapoints (critical data). Trialists began to conduct central reviews of the database and to contact sites or make an on-site monitoring visit if the central review showed an apparent need. Risk-based monitoring was enshrined in International Council for Harmonisation of Technical Requirements for Pharmaceuticals for Human Use (ICH) GCP E6(R2) in 2016 and amended in 2018,^3^ with all trials encouraged into this monitoring strategy.^4–6^ In risk-based monitoring, the monitoring activities are focussed on preventing or mitigating risks to data quality that are both important and likely. These must be risks to processes critical to human participant protection (rights, safety and wellbeing) or to trial integrity. Rather than monitoring broadly all aspects of the trial, monitoring is directed at these pre-defined risks to the trial and also to risks which become apparent during the trial. Risk-based monitoring often starts with central monitoring which is monitoring performed in a location away from the investigator site and often at clinical trial unit/sponsor offices. It involves an evaluation of accumulating data (or lack thereof), performed in a timely manner and supported by appropriately qualified and trained persons.^7^ This central monitoring is followed by escalation to an on-site monitoring visit, if concerns about a site warrant it. Some element of source data verification may be mandated, but often only for a small selection of data or participants. Monitoring is applicable to all trials, with clinical trials of investigational medicinal products tending to have a higher risk and therefore requiring more extensive monitoring.

It is particularly the terms and processes of central monitoring and data cleaning that are confused. Table 1 defines data cleaning and central monitoring. As an example, a data cleaning activity might be sending out a list of queries for site teams to resolve, whereas a related central monitoring activity might be looking at query resolution rates across different sites and escalating, if a certain percentage of queries have remained open for 6 months or more. Table 2 contrasts these terms.

**Table 1.** Definitions.

**Data cleaning:** Data cleaning addresses problems with data such as incomplete, invalid or inconsistent data. When data are entered, most databases have some automated checking of data and flagging of problems. On a regular basis or maybe before data monitoring committee (DMC) meetings, central trial team members run checks on the participant data and query any strange or required values with sites. Before any interim or final analysis, these processes will be repeated. These are all data cleaning activities. They happen often in the course of a trial. The main action is sending out data clarification requests.
**Central monitoring:** Central monitoring is looking to centrally identify any issues with trial conduct such as inadequate processes or procedures not being followed through a lack of clarity in the protocol or active fraud. Looking through centrally held data by site, to discover odd patterns or features in the site’s data (e.g. missing treatment data) or unacceptable data activity (e.g. digit preference in white blood cell level), during the trial, at times specified in the trial’s trial monitoring plan, is best called ‘central monitoring’. This may result in data queries to sites or may provoke dedicated communication with sites or an on-site monitoring visit. Central monitoring results are an indicator of the quality of a trial and show due diligence. Any issues found during central monitoring should be followed up by contacting the site and may also result in actions such as the delivery of (re)training or the making of an on-site visit. Central monitoring need only be repeated periodically, the period depending on trial parameters such as the duration of treatment and recruitment rate and on the assessment of risk. Sometimes central monitoring is done across sites, comparing data between sites to show differences. In some instances, this may be done across trials run from the same organisation. Central monitoring can include review of trial management data such as records of protocol deviations.

**Table 2.** Data cleaning and central monitoring similarities and differences.

	Data cleaning	Central monitoring
Purpose	To ensure the data are accurate and complete	To ensure the trial is being run according to the protocol
Scope	Individual questions and participants	Site level or across sites and trials
Evaluates	Issues with data recording or data entry	Issues with processes
Likely actions	Send out a data clarification request	All or any of Contact with site Site (re-)training On-site visit
Mutual benefit	Good data cleaning leads to fewer monitoring actions	Can include consideration of the success of data cleaning, for example, using a metric of the percentage of data queries outstanding at 2 months
Frequency	Soon after data entry	Periodic, depending on the risk
Data monitoring committee (DMC) and analyses	Cleaning activities may be increased before each interim and final analysis	Periodically performed but may be also carried out before each DMC review and analysis
Specification	In data management plan	In trial monitoring plan
Summary measure of effectiveness	Often counts or percentages, for example, of non-missing variables or case report forms or variables out of range	Can be summarised as ‘quality tolerance indicators’ to give a single value or small number of values to express the current quality of the trial
Funding	Often bundled in with trial staff time	Sometimes encompassing dedicated staff (monitors)

Central monitoring may be split into many tasks which are completed across time in a rolling pattern, for example, serious adverse events in week 1, protocol deviations in week 2, case report form return rate in week 3, serious adverse events in week 4 and so on. Our term ‘repeat central monitoring’ is referring to the re-running of the same central monitoring task(s).

Without a clear understanding of data cleaning and central monitoring, the trial team and site staff may spend time and effort inappropriately or wastefully. If these activities are not separated, they can each occur at the wrong time: data cleaning too rarely and central monitoring too frequently. Data cleaning needs to happen often. It is easier to clarify, correct or locate previously missing or out-of-range datapoints when the query is asked close in time to when the data were collected. Data cleaning needs to be done often so that the data are as good quality as possible for central monitoring to be effective. Central monitoring is most effective on cleaned data, otherwise teams will focus on individual data errors rather than required process changes, or an incorrect process may be missed due to poor quality data. Repeat central monitoring needs to happen periodically. Trial teams need to have the time and capacity to consider the central monitoring findings and take appropriate action. Action will take time. The interval between running repeat central monitoring reviews needs to be long enough so that site staff who action the central monitoring findings have had time to do so. The actions do not need to be complete but some work needs to have been done. In most trials, daily central monitoring is not viable. Central monitoring needs to happen to pick up real, systemic problems, not momentary blips. Similar to interim analysis being done at planned times so as not to inflate the chance of a positive finding, central monitoring repeated daily, for example, for all except fast recruiting short duration primary outcome trials, will find problems that are not real or that are transitory and do not require extra input. Resources are required for each of data cleaning and central monitoring. Appreciating their benefit to the trial is a part of resourcing.

The quality of the trial will suffer if the differences between data cleaning and central monitoring are not well appreciated. If they are not separated, then either or both could be done inadequately. By considering them as one, it can feel like enough is being done. If they are not done separately, then it may be that a risk for a trial is not adequately covered. Central monitoring is often considered in a risk-based framework relating to the written risk assessment of the trial. Though data cleaning protects the integrity of the trial and may be based on risk (e.g. variables considered critical to trial completion may be cleaned more often), it is not framed in a risk-based way. Therefore, there is scope for a risk noted in central monitoring to be part covered by a data cleaning task, resulting in the risk not being adequately covered. Data cleaning is done on individual participant data and central monitoring is carried out on all available (and missing) data at one site. Data cleaning will not be so effective done at site level and central monitoring may miss a risk if it is done per individual participant.

If the research community cannot be clear on language, it is difficult to discuss best practice or, importantly, define high-quality methodology projects to determine evidence-based improvements to approaches across trials.

In conclusion, it is important to correctly define data cleaning and central monitoring in order to communicate the conduct of a trial, to ensure adequate risks mitigation and to ensure that the data are appropriately corrected. This commentary starts this discussion.
